# Extraordinary issue: Coronavirus, crisis and communication

**DOI:** 10.1177/1329878X20960300

**Published:** 2020-11

**Authors:** Adrian Athique

**Affiliations:** University of Queensland, Australia

It does not need to be said that we live in extraordinary times. The majority of the world’s population are living under some form of movement restrictions, and our daily work, sustenance and sociability have been almost entirely mediatized. As scholars of media and communication, there are critical developments in play that deserve our attention and our voice, and *Media International Australia* is pleased to offer this first selection of articles and commentaries from our Extraordinary Issue on Coronavirus, Crisis and Communication.

There has never been a time in which media systems have been able to convey such detailed and universal coverage of an historical event in real time, with the added capacity to keep us all in touch and to give us a voice too. At the same time, the vast social narrative of this pandemic has been visibly characterized by confusion, misinformation, disinformation, charges of conspiracy, cover-ups and multi-vocal denials. In among this, public health experts have been thrust into the limelight, as they try to guide nations and their leaders, as have economists seeking to secure our futures in the absence of a machine that was never intended to stop.

Media experts, too, have their part to play in helping people to make sense of the times that we are living through. With this in mind, this extraordinary issue of MIA offers a selection of contributions that address the critical role of communication during the ‘first wave’ of the COVID-19 pandemic in the first-half of 2020. Given the scale and scope of this global pandemic, and its intense mediation, this selection will be followed by a concurrent issue, extending our account into other facets, topics and locations of communication under pandemic conditions.

Where we are now is already very different from where we were when these articles and commentaries went to press, and even further from when the original submissions were produced. Given the urgency and immediacy of applying communications expertise at a time of crisis, we invited contributors to work outside of the normal timeframes of academic publishing in our field, inviting them to share immediate ideas and concerns and to present ongoing research much earlier in the working process than they would in less extraordinary circumstances. Thus, in many cases, the final and definitive account of these topics and research projects is yet to come. Nonetheless, our early engagement here with the consequences of the pandemic reflects the generosity of our contributors as well as the salience of our field.

In this instalment of our extraordinary issue, we present three sections of full articles and shorter commentaries. The first of these, ‘pandemic as data’, examines the role of digital architecture as a medium of record of the public experience as well as a mechanism for interventions in public health regimes. The second section, ‘pandemic, health and affect’, provides commentary on the interaction between health communication and the emotional and cognitive experiences of the pandemic as a state of crisis. The third and final section of this extraordinary issue offers a selection of international perspectives that examine the pandemic as it unfolds under the conditions of a suspended globalization, where Coronavirus manifests as a global crisis mediated through national, and sub-national, agencies.

## The flight of black swans

Baked into the social logics of our digital architecture is an iterative triad of free speech, mass surveillance and predictive calculus – designed to provide simultaneously a universal public sphere and an unprecedented mechanism of managerial control. Consequently, as a BBC commentator on the Coronavirus observed, ‘we thought digital technology would keep us safe’ – this being the implicit tradeoff between intrusive communication technologies and personal well-being. From the apex perspective, the cacophony of the digital demos and the diminishing of state hegemony over public communication was the tradeoff for nourishing the digital harvest of public opinion for strategic gain. In this context, it becomes axiomatic that digital solutions are touted as the strategic remedy for all manners of social ills, economic deficiencies and emerging risks.

Our awareness of the dangers of this approach has been growing, and it has become particularly acute in the circumstances we now face. Thus, the COVID-19 pandemic appears simultaneously as a biological threat to humanity and as a crisis for the very ethos of digital society, where historical experience is proving more instructive than futuristic prediction, where social consensus is proving more effective than automated surveillance and where the central role of digital communication alleviates social dislocation, but amplifies hysteria. Despite sustained efforts to naturalize the affordances of the digital as functional and technical – and thereby agnostic and inevitable – our recourse to the digital has been effectively shaped by a series of crises in the 21st century.

This series of seemingly ‘black swan’ events at the global scale were all eminently predictable in nature, if not in their specific timing, and each of them presented wicked problems that appeared to require complex responses using the preeminent technology of our time. At the turn of millennium, it was the terror attacks of 9/11 which legitimized the implementation of mass citizen surveillance, and novel data-sharing relationships between technology companies and state agencies. It was the Global Financial Crisis at the end of the 2000s that drew our attention to the systemic risks created by the increasing velocity of finance and to the undue complexity facing economic managers. A decade later, the global pandemic of COVID-19 has presented us with the greatest social, behavioural and logistical challenge since the 1940s. Given our path dependencies, it is of no surprise that digital communication technologies have been absolutely central to our collective response.

## Predictive calculus

There was much fanfare in 2010 surrounding Google Flu trends and its claimed capacity to predict outbreaks of disease by drawing upon its panoptic view of search terms and identifying epidemiological trends through pattern recognition (Preis and Moat, 2014). Things went quiet as it became apparent that recording one outbreak in detail through proxy inputs does *not* provide predictive capacity (Lazer et al, 2014). Parallel efforts to predict novel disease outbreaks ran up against the sheer volume of virological organisms, the multiple variables involved in zoonotic transfer and in human transmission and disease. By 2017, it was conceded that such events were both inevitable *and* unpredictable (Yong, 2017).

This dilemma, the ‘white swan’, strikes at the heart of an ascendant numerological privilege. Nonetheless, in the first 3 months of the current pandemic, data modelling was the only *authoritative* influence on policy makers, even where such work had to be based upon limited knowledge of the pathogen or the diseases that it causes. Imperial College London (ICL), who raised the alarm early in January, were on the strength of their reputation and data, the first people outside of Asia to cut through the ‘do not be alarmed’ messaging and start saving lives.

The ICL data were based upon early assumptions about the virus, many of which turned out to be false. But even without reliable inputs, the deductive capacity of human beings had greater utility than predictive technologies, with the caveat that it only gained credence when dressed up in numbers. Once the mechanics of a virus are known, it will become effectively predictable, but the insurmountable problem remains that the carriers of the virus are far more complex organisms, and can thus only be *intuitively* predictable. The lesson to be learned from our failures, and from our successes, is that prediction can never be a substitute for preparation. It is far more important to have the necessary procedures and stockpiles in place than to predict the point of occurrence.

## Perpetual traces

Prediction is not, however, the only potential of granulated digital systems. In less than two decades, mobile architecture, device dependency and bureaucratic imposition have given us the most extensive regime of surveillance ever known. This in itself, predisposed, the ‘trace, test, isolate’ doctrine that was widely touted as the SMART alternative to welding people inside buildings. Singapore, with its much-envied technocratic regime and well-disciplined population, was a natural leader in developing mobile-based tracking.

But, as Terence Lee and Howard Lee (2020) argue in this extraordinary issue, the TraceTogether software failed to prevent an outbreak of Coronavirus – not only because the Bluetooth environment was not conducive to this re-purposing – but also because it was predicated upon a certain normative Singaporean, and not on the crowded and impoverished transient population that the government has itself conspired to render invisible. Nonetheless, the subsequent implementation of a QR code and bracelets regime demonstrates an ongoing commitment to the vision of SMART cities, as much as a functional test of the fundamental premise of a digital civic future.

In his article, Gerard Goggin (2020) goes on to extend the Singaporean experience with a comparative account of how the Australian federal government, among others, latched onto the promise that perpetual contact-tracing through phone apps would allow an ‘acceptable risk’ environment for everyday economic life to resume, thereby bolstering the surrender-and-suppress doctrine favoured by economic managers. This comparison allows Goggin to unpick the ways in which differing civic norms and regimes of privacy were negotiated, and how the perceived tradeoffs between public health management were articulated in each case.

As we now know, by the time that this digitally enabled resumption of normality was attempted, the United Kingdom had to concede that their app just did not work, while Australia has also had to adjust to the evident failure of the CovidSafe ‘sunscreen’ in Melbourne. Around the world, Black Lives Matter (BLM) protests, anti-mask protests, 5G conspiracy demonstrations, illegal parties and skiing trips all served to demonstrate the limitations of automated governance. Surveillance, without the threat of violence, is no substitute for compliance. Even in the origin case of China, the tragedy in Wuhan was a powerful reminder that the vast investments in surveillance and techno-authoritarianism would have been far better spent on addressing basic failures in public hygiene and sanitation. The lesson here is that controlling a virus is not like controlling people, which is hard enough. A virus does not carry a phone.

## Free speech

At best, digital solutions have been used to provide ontological security, but soap and masks have proved to be more effective containment technologies. Nonetheless, the multi-purpose architecture of our interactive media systems puts communication at the centre of government action, if not education policy. A negative consequence is that our managerial culture is overly dependent upon data-driven decision-making and predisposed towards an app-based solution for almost everything, what Morozov (2014) has called the ‘solutionism’ of our times.

A positive consequence is that all of us have the personal capacity to assimilate and interpret vast amounts of information at unprecedented range, which is an imperative for citizen negotiating a fast moving virus in a world moving at speed. In the public domain, the many-to-many configuration of the Internet and the echo chambers of social media platforms are fundamentally different from the mass media systems through which our response to the AIDS pandemic was enacted 30 years ago (Edgar et al., 1992). In that case, a dire and officially orchestrated warning was broadcast throughout society, with relatively minimal noise from gainsayers and bigots, and a remarkable institutional consensus among public health professionals, journalists and politicians.

By contrast, as a rich and detailed contribution from Axel Bruns et al. (2020) in this extraordinary issue demonstrates, the current pandemic has been characterized by a bewilderment of mixed-messaging, counter-arguments, motivated activism, geopolitical conflicts and conspiracy theories. The latter range from the officially sanctioned allegations of laboratory release, to bizarre claims around 5G, and irrational insistence by so many that the Coronavirus is not dangerous or does not even exist. This datalogical research illustrates in high fidelity the origins and network dynamics of 5G COVID-19 conspiracy theories across the major platforms of the global communications architecture.

Taking the same example, Rowan Wilken James Meese and Jordan Frith (2020) provide a critical interrogation of how these networks of dissemination interact with the very same digital infrastructure that they are seeking to attribute this human calamity to, thereby unpicking the confused logics of our times. Just like everywhere else, these conspiracy theories were used to justify the parties taking place in my suburb throughout the first lockdown. The universal import is that, in critical ways, the libertarian ethos of free speech, participatory journalism models and the emergent modes of political populism enabled by social media platforms have all shaped the temper of social responses to the pandemic.

For its part, public health communication has been bedevilled throughout by political constraints and incomplete or incorrect advice on the nature of the beast. From limited human transmission to high contagion. From surfaces to airborne transmission. From immunity passports to reinfection. From mild illness for most to protracted recovery and lasting organ damage for many survivors. From childhood immunity to widespread outbreaks in schools. Consequently, the return of public experts to the press podium in March 2020 has been followed by a descent into vilification, partisan politics and, most critically, denial. This has been true both nationally and internationally, with Altman Peng’s (2020) compelling account of the hospitalization of Boris Johnson providing a useful insight into the geopolitical discourse of leadership in the time of COVID-19. In retrospect, the British Prime Minister’s insistence on ‘shaking hands with everyone’ appears as daft as the bravado of other, mostly male, world leaders seeking to calm the public with their own invincibility.

## Public health and affective disruption

Such displays of denial are a natural human response to complex and intangible threats, and even more so to something that so powerfully disrupts the patina of normality through which our life courses are perceived. As Elizabeth Stephens notes in her contribution to this issue, the desire for a ‘snapback’ to normality is an overwhelming, but problematic, impulse in a situation which uniquely disrupts everyday life everywhere (2020). Having derided the ‘cancel culture’ of which he is the outstanding exemplar, the US President has twittered the demise of the Coronavirus repeatedly since February. The virus itself, unfortunately, remains impervious to being blocked from anyone’s personal reality. The lesson here is that ignorance shares many characteristics of a viral disease, and the practical question is what doctors of communication can do about that. Do we control that virus or does it control us?

Addressing the interaction of social media and public health communication directly, Stephanie Baker’s (2020) commentary illustrates the complexities of conceptualizing and assessing the potential harms caused by misinformation, disinformation, contestation and confusion in messaging around the pandemic. Having taken on themselves the onerous concept (and dubious sovereign privilege) of deciding what content is harmful or not to the public, the major social media platforms are making decisions around what is just comment on the ever-evolving facts of COVID-19 and making critical judgements about harm in real time. Since the concrete proof of harm is only readily available in a small number of cases, as with people drinking bleach, this larger responsibility is almost impossible to take carriage of. In a field of unknowns, as Stephanie notes, such interventions can only be ideological.

Nonetheless, there is already a powerful sense of scale of harm arising from the pandemic, both epidemiological and in the attrition of emotional and mental health, a concern which is often problematically linked to economic rather than pastoral remedy. By contrast, Phoebe Elers and Mohan Dutta (2020) take up exemplars of more avowedly affective strategies for supporting societies in the time of pandemic, analysing the mobilization of the concept of ‘kindness’ in New Zealand, India and Singapore. Here, kindness has been deployed as an entreaty for citizens to undertake acts of altruism and demonstrate togetherness towards the disadvantaged and/or marginalized communities who are suffering the greatest collective harm from the economic and logistical impact of the pandemic shutdowns.

In their critique of public kindness, Elers and Dutta point out how such strategies of kindness outsource the responsibilities of governments towards disadvantaged citizens to the general public, and too often obfuscate a substantive discussion about the systemic basis of precarious lives. This begs us to question whether such kindness is a civic duty only for the duration of the disaster, warranted as long as a risk to our own health is implied by the desperate conditions of others. Are these acts of kindness, along with public demonstrations for health workers, primarily displays of a collective desire to be rescued by the sacrifices of others, or are they evidence of a collective goodwill that may lead us towards what Elizabeth Stephens and others are calling the ‘new normal’?

As time passes by, are these public displays of sociality degenerating into blame, suspicion and, when the new normal arrives, will we have learned the most critical questions about human interdependency and about who is considered essential and who is not? As Rachel Mayer and Blaise Murphet (2020) comment in the same section of this issue, there are powerful lessons available from other recent disease outbreaks such as Ebola, highlighting the critical role of institutional health communication and community engagement in navigating major health crises. In some respects, the underlying point is that is the speed, scale and scope of COVID-19 that makes it appear to us as a disaster without precedent. Yet, outbreaks of infectious diseases are ongoing realities of human experience, and there are many available lessons to be learned, not only from the similarly scaled influenza in 1918, but also from more recent disease outbreaks such as Ebola and SARS.

One of these lessons is that trust is an essential factor in mitigating epidemics, and that it is easily undermined by political conflict and institutional incoherence and incompetence. Another lesson is that communication is a core business of public health. Taking these lessons to heart, Mayer and Murphet identify the fieldwork experience of humanitarian workers and the sociological knowledge of communications scholars as necessary agents of reform for the world’s major public health institutions and, equally, for national and state health officers who have been thrust into the public spotlight for months without respite. Gabi Mocatta and Erin Hawley (2020) position environmental communication as another instructive domain, not least due to its formulation as a ‘crisis discipline’ engaging simultaneously with policy makers, scientists, corporate interests, and the general public.

This orientation towards worldwide crisis positions environmental communication models as strategically apt in the time of Coronavirus, while also highlighting the sensory experiences of anxiety and worry that such vast challenges instil among all of us. In the public domain, Mocatta and Hawley note that the early displacement of omnipresent environmental concerns by the Coronavirus outbreak has since evolved into a new discursive mode where environmental disruption and the pandemic event are being firmly linked. In the Australian experience, of course, the twin disasters of conflagration and contagion in 2020 have overlapped in such a way that – both practically and emotionally – the ensuing state of disaster and state of mind have been protracted experiences.

Consequently, one immediate lesson to take from this set of commentaries on pandemic, health and affect is that a critical understanding of structures of feeling becomes a vital input to effective communication and ensuring public health. Looking forward, exhausting as it is, we can also see that our evolving sense of the emotional registers of the pandemic – of harm, of worry, of kindness – is a powerful instruction in the sociality of the new normal to come.

## Global perspectives

The juxtaposition of environmental and epidemological crisis illustrates the global scale of both, toughing each and every corner of the earth in ways that even the largest political events do not. The scale of this global experience is hard enough to grasp in itself, and especially so at a moment when a world of unprecedented mobility has come to a shuddering stop. I myself experienced the first 2 months of the COVID-19 in four different countries, taking some 20 flights before the first wave of lockdowns began and international borders closed. Six months later, with airline fleets parked out in deserts, the prospect of such mundane itinerant velocity has receded into a seemingly distant future or an anachronistic past.

After a decade in which economic contagion has diminished the spell of globalization as an ethos of human ambition, the biological contagion of Coronavirus has highlighted both the risks of global mobility and the human toll of restricting it. I am a citizen of two countries, neither of which I can reach, although I am both privileged and grateful not to be stranded, either offshore or onshore, in desperate straits as so many people currently are. The sundering of the global has been reproduced down to the state, metropolitan and suburban boundaries as borders appear everywhere. Thus, as much as the pandemic is a universal and global human experience, it is also subjectively and administratively constrained with the parameters of nation states and their constituent parts.

Consequently, there are critical variations of experience across countries with different political systems, historical experiences and cultural norms. The global pandemic is rendered in important ways as a geography of national crises in a world of suspended globalization. The international dimension of this dynamic is played out in the statistical contest of which nation is best managing the pandemic death toll, the economic carnage or the governance of lockdown regimes, not to mention the quickening race for a COVID-19 vaccine. It seems apt, then, to end the first instalment of our extraordinary issue by bringing together commentaries on the pandemic from countries at once familiar and now impossibly distant for most of us. To begin with, Rodrigues and Xu returns our focus to the circus of fake news and misinformation, and the challenges of media governance arising from the interaction of social media and a state of pandemic.

By providing a comparative account of how these dynamics played out in India and China in the early stages of the pandemic, Rodrigues and Xu (2020) sketches the strategic logics of social media governance in the two most populous countries of the world. Despite being founded upon very different political and philosophical regimes, both countries face enormous challenges in managing an epidemic among vast and concentrated populations, both have large and highly active social media domains and, at the time of the outbreak, both countries were already subject to increasingly authoritarian political regimes. It seems likely that the legitimacy of these trajectories will be set by how effective such authoritarian modes of governance prove to be in managing the pandemic.

In the Philippines, which recorded some of the first cases outside of China in January 2020, an established (albeit controversial) authoritarian mode of governance was similarly available to enforce both comprehensive metropolitan lockdowns and the massive repatriation of a precarious expatriate workforce from across the world. Shifting our attention away from the governance of misinformation to the ‘traditional’ role of journalists as informants and educators, Jan Michael Alexandre Bernadas (2020) comments on the viral role being played by journalists in the Philippines in safeguarding public health, at a time when the relationship between political authority and these agents of civil society has itself been at a point of crisis.

Writing of the Russian experience, Svetlana Bodrunova (2020) discusses the role of citizenry in the constitution of socially mediated ‘contributive action’ following the onset of the pandemic. This account provides a counterpoint to our widespread concern with social media as an ‘infodemic’ of falsehoods by considering instances of civic and political engagement fashioned in online spaces during the Russian lockdowns of April to June 2020. These engagements have encompassed both patriotic storytelling and public protest, expressed variously through the aesthetics of art and politics. As ‘grassroots’ movements, these online campaigns sought to fashion and nurture online publics under curfew, eventually prompting both repression and recognition from bodies of the state. These instances encourage us to think of the publics of the pandemic in ways other than their credulity or compliance.

Continuing with a focus on citizenship under crisis, Giang Nguyen Thu offers a final commentary on the ways in which communal and historical experiences of conflict, communism and contagion allowed for a highly effective community response to the first wave of pandemic in Vietnam (2020). This predisposition was subsequently channelled into state propaganda that sought to correlate this early success with legitimacy of the party. Thus, a successful public response is put to the purpose of normalizing a political system, as the government positions itself as the key agent in ensuring a return to normality. Thus, akin to the ‘snapback’ in Australia, the party line is that the optimum outcome of this crisis will be where the new normal is the old normal.

These global commentaries are useful not only in balancing our sudden national isolations, or for comparing the contrasting political systems under which public health challenges must be addressed. Rather, they serve to demonstrate the scale of the social and political ramifications of the Coronavirus, and teach us important lessons about the geography of this global pandemic. These countries neighbouring China were not, as anticipated, the first dominoes to fall in the onset of Coronavirus. They prompted swift and largely effective pandemic responses, informed by their awareness of proximity and prior experiences of epidemics such as SARS. Instead, it was the more distant regions of Europe and America, complacent to the vectors of global mobility that descended first into disaster. From where we are now, as a series of ‘second waves’ has afflicted all of these countries, some of them very badly, these early lessons will nonetheless remain instructive, even as our attention to the geography of the pandemic turns towards the political economy of vaccination.

## Communication as continuing research

Taking account of our experience thus far, it would be fair to say that, outside of virology, communication may well prove to be the most central field of expertise in countering this pandemic. Thus, these vital contributions to the first part of our extraordinary issue begin to demonstrate both the centrality and complexity of communications research amid the immediate impact of Coronavirus. In the second part to follow, we will turn our attention towards the experiences of children, of workers in the creative industries, and the implementation and expansion of remote presence and digital logistics in every corner of life.

As our own daily work continues – variously through video conference, emails, tweets and blogs – the learning by doing that is intrinsic to our field is developing in important ways. As such, we are pleased to also include here a number of excellent general articles, from Stuart Cunningham and Alexa Stuart (2020), Alan McKee (2020) and Steven Maras (2020), that detail ongoing work on the media industries, media education and within the Australian and New Zealand Communication Association. For our part, both Craig Hight and myself as editors would like to thank all of the contributors, the MIA board and production staff working under novel conditions at Sage in India for making this extraordinary issue happen in these extraordinary times.
